# Absolute structure determination of Berkecoumarin by X-ray and electron diffraction

**DOI:** 10.1107/S2053229624003061

**Published:** 2024-04-10

**Authors:** Daniel Decato, Lukáš Palatinus, Andrea Stierle, Donald Stierle

**Affiliations:** a Chemistry and Biochemistry, University of Montana, 32 Campus Drive, Missoula, Montana 59812, USA; b Institute of Physics of the CAS, Na Slovance 1999/2, Prague 19200, Czech Republic; University of Delaware, USA

**Keywords:** crystal structure, absolute structure determination, electron diffraction, microED, dynamical refinement, Berkecoumarin, chromenone, coumarin, natural product

## Abstract

X-ray and electron diffraction methods independently identify the *S*-enanti­omer of Berkecoumarin [systematic name: (*S*)-8-hy­droxy-3-(2-hy­droxy­prop­yl)-6-meth­oxy-2*H*-chromen-2-one]. Isolated from Berkeley Pit Lake *Penicillium* sp., Berkecoumarin is a natural product with a light-atom com­position (C_13_H_14_O_5_) that challenges in-house absolute structure determination by anomalous scattering. This study further demonstrates the utility of dynamical refinement of electron-diffraction data for absolute structure determination.

## Abstract 2

The absolute structure of Berkecoumarin, a natural product from a Berkeley Pit Lake *Penicillium* sp. is reported from both X-ray and electron diffraction data.

## Introduction

**Figure 1:**
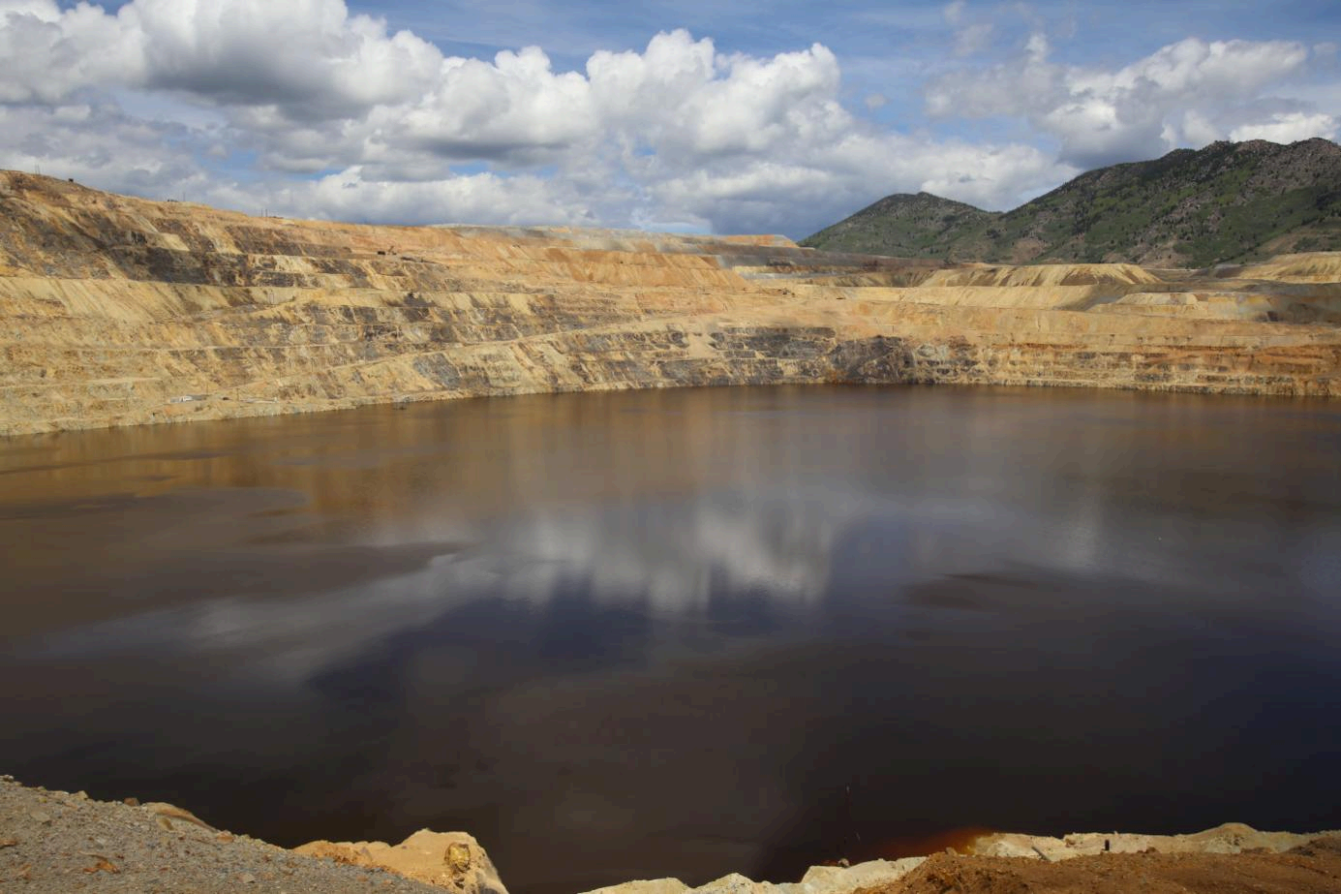
Berkeley Pit Lake.

The Stierle lab has dedicated nearly 30 years to investigating extremophilic fungi derived from an acid mine waste lake in Butte, Montana. Situated within the largest United States Environmental Protection Agency Superfund site, the Berkeley Pit Lake system encompasses an abandoned open-pit copper mine, measuring 1500 feet in depth and one mile across. As infiltrating groundwater inter­acts with the pit, rich veins of pyrite and other minerals dissolve, resulting in acid generation. The Pit holds nearly 35 billion gallons of water, with a daily inflow of >2.5 million gallons, characterized by an acidic nature (pH 2.7) and contamination with elevated metal sulfates (*e.g.* 1000 ppm iron, 150 ppm copper, and 600 ppm zinc) (Gammons & Duaime, 2006 ▸) (Fig. 1 ▸). 
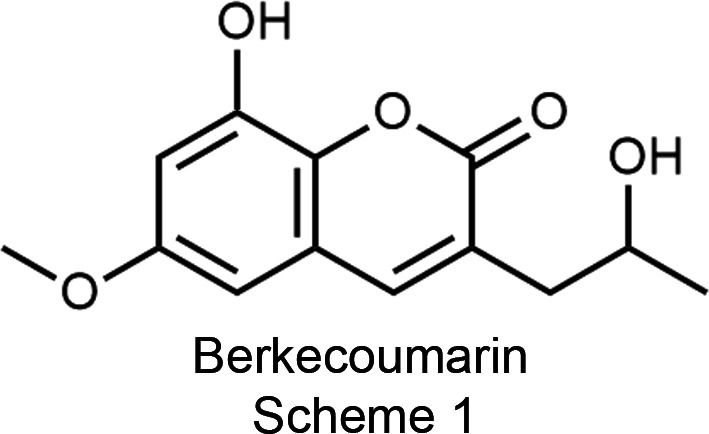


While research on the chemistry and potential remediation strategies of the Berkeley Pit Lake spans almost 40 years, the microbial ecology was neglected until the Stierles began their investigation of the secondary metabolites of the resident fungal extremophiles. Although the Berkeley Pit was assumed to be too toxic to support life due to the low pH and high metal content, the Stierles, in collaboration with Grant Mitman, isolated over 40 fungi, protists, algae, protozoans, and bacteria from its water and sediments (Mitman, 1999 ▸). Despite the toxic conditions for conventional aqua­tic biota, the Pit Lake system provides an ideal environment for extremophiles, potentially fostering new species to produce unique secondary metabolites. The challenge of natural products drug discovery lies in devising methods to target the bioactive compounds within these organisms.

In 2004, the Stierle lab isolated Berkecoumarin, from a Berkeley Pit Lake *Penicillium* sp. (Stierle *et al.*, 2004 ▸). Initial analysis using high-resolution electrospray ionization mass spectrometry revealed the mol­ecular formula as C_13_H_14_O_5_. A series of NMR studies facilitated structural elucidation, as depicted in Scheme 1. Berkecoumarin is among the rare 3-alkyl-6,8-di­oxy­coumarins sourced from fungi, with another instance being 3-hy­droxy­methyl-6,8-di­meth­oxy­coumarin from *Talaromyces flavus* (Ayer & Racok, 1990 ▸).

The bioactivity of Berkecoumarin has been explored. One study demonstrated the ability of Berkecoumarin to traverse cell membranes and inhibit caspase-3, suggesting a potential neuroprotective effect post-stroke (Stierle *et al.*, 2017 ▸). Des­pite previous studies, the absolute configuration of Berkecoumarin remained elusive. In this article, we present the absolute structure of Berkecoumarin, employing both X-ray diffraction methods and dynamical refinement of microcrystal electron-diffraction data.

## Experimental

### Metabolite generation and isolation

The collection, extraction, and isolation of Berkecoumarin has been described previously (Stierle *et al.*, 2004 ▸).

### X-ray data collection and processing

Crystal data, data collection, and structure refinement details are summarized in Table 1 ▸. All non-H atoms were refined with anisotropic displacement parameters. It was possible to identify H-atom positions from the difference Fourier maps. H atoms bound to O atoms were placed and refined. Those bound to C atoms were placed in geometrically calculated positions and refined using a riding model. Isotropic displacement parameters of the placed H atoms were fixed at 1.2 times the *U*_eq_ value of the atoms to which they are linked (1.5 times for methyl groups).

### MicroED data collection and processing

Very fine needles of Berkecoumarin, obtained by slow evaporation of a deuterated chloro­form solution, were ground, then deposited on a pre-clipped continuous carbon film on Cu 200 mesh (Ted Pella 01840). The grid was then plunged into liquid nitro­gen, and transferred under cryogenic conditions to the microscope. Continuous rotation electron-diffraction data were recorded using a Thermo Fisher Scientific Glacios Cryo Transmission Electron Microscope (operating at 200 keV) equipped with a CETA-D detector. Automated tilt series data collection was carried out using *Leginon* software (Cheng *et al.*, 2021 ▸). A total of nine dif­frac­tion data sets were collected under parallel illumination conditions and under cryogenic temperature (≃ 105 K). After visual inspection, four data sets were removed due to poor quality, leaving a total of five data sets for data reduction and further analysis. A 20 µm condenser aperture was used during data collection, resulting in a ≃ 0.6 µm diameter beam on the specimen.

### Dynamical refinement processing

The data were processed by the program *PETS2* (Palatinus *et al.*, 2019 ▸). The processing revealed high mosaicity for all five data sets considered, sometimes accompanied with reflection splitting. These traits are unfavorable for dynamical refinement, which is, in its current implementation, based on the assumption of a perfect crystal. In the case of imperfect crystals, the results of the dynamical refinement tend to be less accurate. However, the absolute structure determination is sufficiently robust to provide reliable results even in these unfavorable cases. Therefore, the best three data sets were selected for the dynamical refinement. Their processing statistics are summarized in Table 2 ▸.

## Results and discussion

### Mol­ecular structure and packing (X-ray)

**Figure 2:**
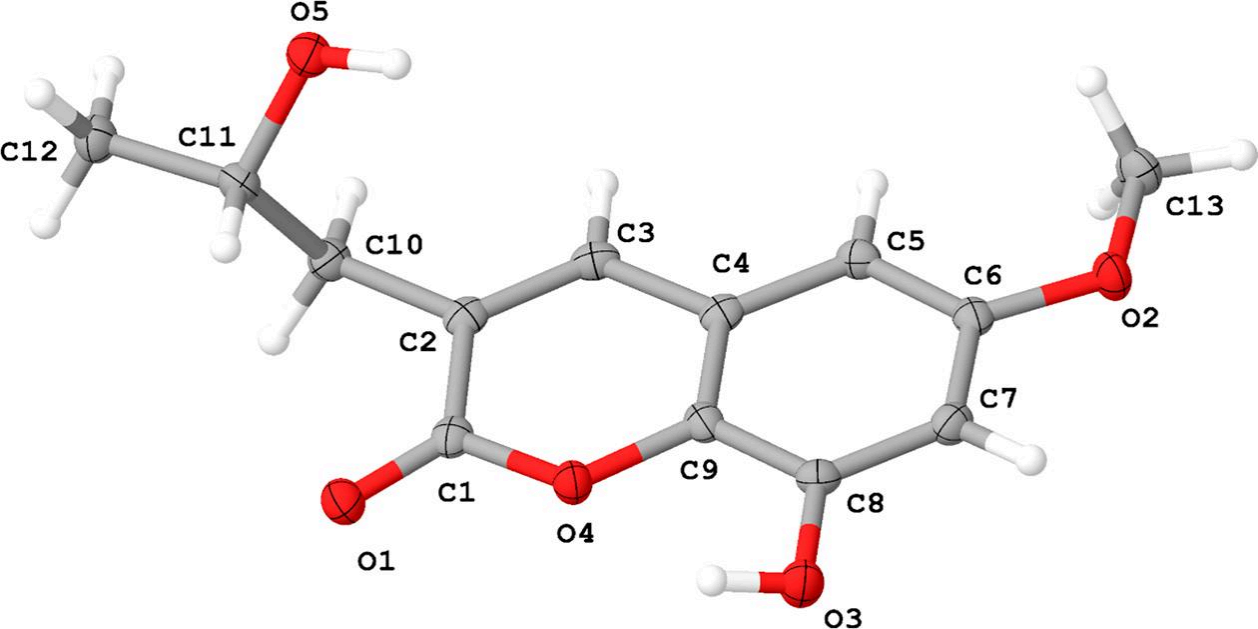
The asymmetric unit of Berkecoumarin with the atomic numbering scheme. Displacement ellipsoids are presented at the 50% probability level.

Small needles suitable for X-ray diffraction were obtained by slow evaporation of a deuterated chloro­form solution of Berkecoumarin. Berkecoumarin crystallized in the ortho­rhom­bic space group *P*2_1_2_1_2_1_ and Fig. 2 ▸ highlights the asymmetric unit.

**Figure 3:**
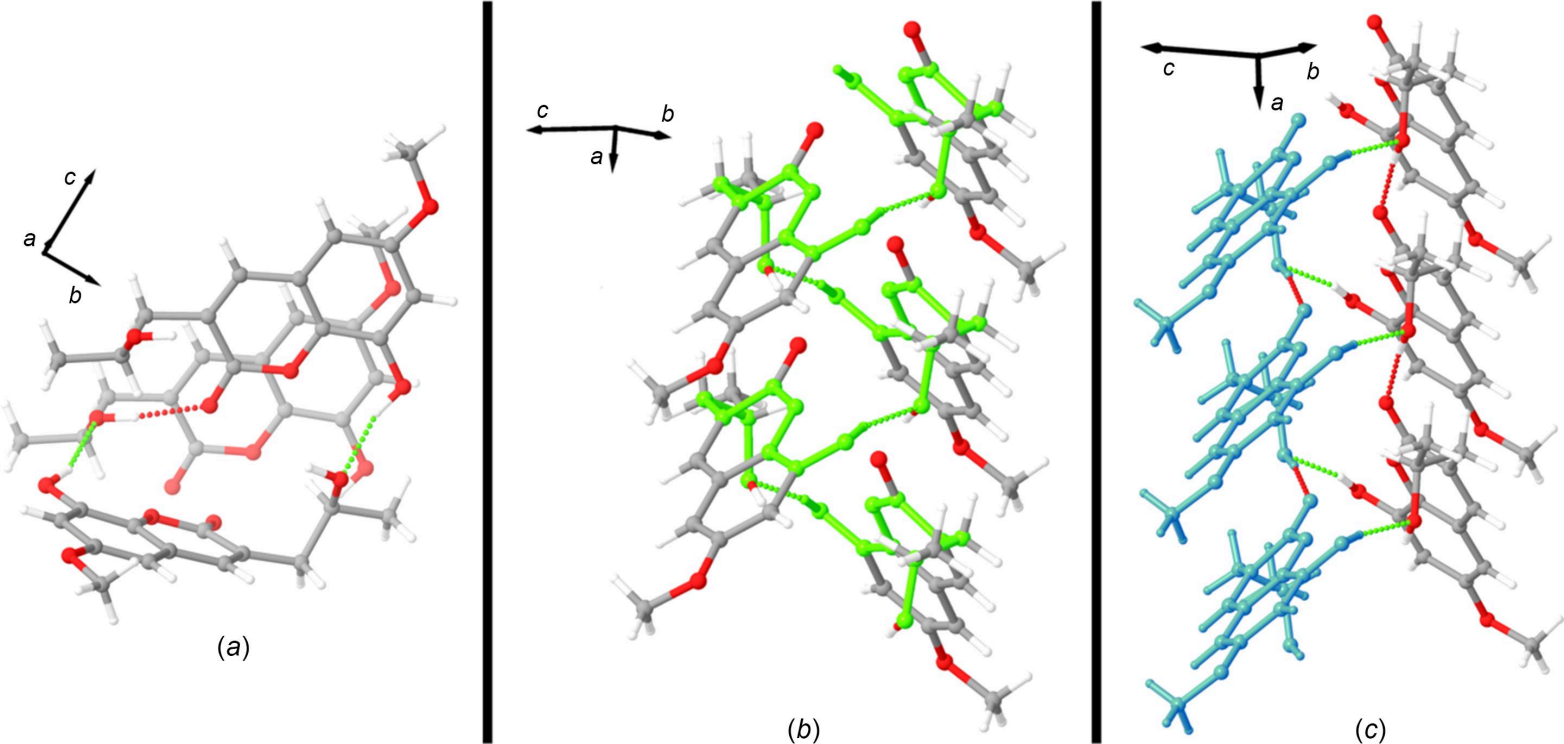
Hydrogen-bond images of Berkecoumarin. (*a*) The two different hydrogen bonds within the Berkecoumarin structure. Hydrogen bonds are displayed by both red and neon green dotted lines. (*b*) Highlighted in neon green is the *C*(10) helical chain formed by the hydrogen bond of the phenolic alcohol group of one mol­ecule to the tertiary alcohol group of an adjacent species. (*c*) The hydrogen bond (red dotted line) of the tertiary alcohol group to the coumarin carbonyl group. Mol­ecules of similar color schemes are ‘above’ each other.

The mol­ecule contains two alcohol groups, each participating in hydrogen-bonding inter­actions [Fig. 3 ▸(*a*) and Table 3 ▸]. The phenolic alcohol group inter­acts with the tertiary alcohol group of an adjacent mol­ecule, with a hydrogen-bond distance and angle for the O3—H3⋯O5^i^ inter­action of 2.723 (3) Å and 161 (4)°, respectively. This hydrogen bond forms helical chains that propagate along the crystallographic *a* axis. This chain described in graph-set notation is *C*(10) [Fig. 3 ▸(*b*)]. The helix is right-handed and seems like a main building block in the crystal assembly. In fact, this helix is further supported by a hydrogen bond between the tertiary alcohol group and the coumarin carbonyl group of a mol­ecule directly above it in the helical column assembly [Fig. 3 ▸(*c*)]. The hydrogen-bond distance and angle of this inter­action (O5—H5⋯O1^ii^) are 2.915 (3) Å and 170 (4)°, respectively.

**Figure 4:**
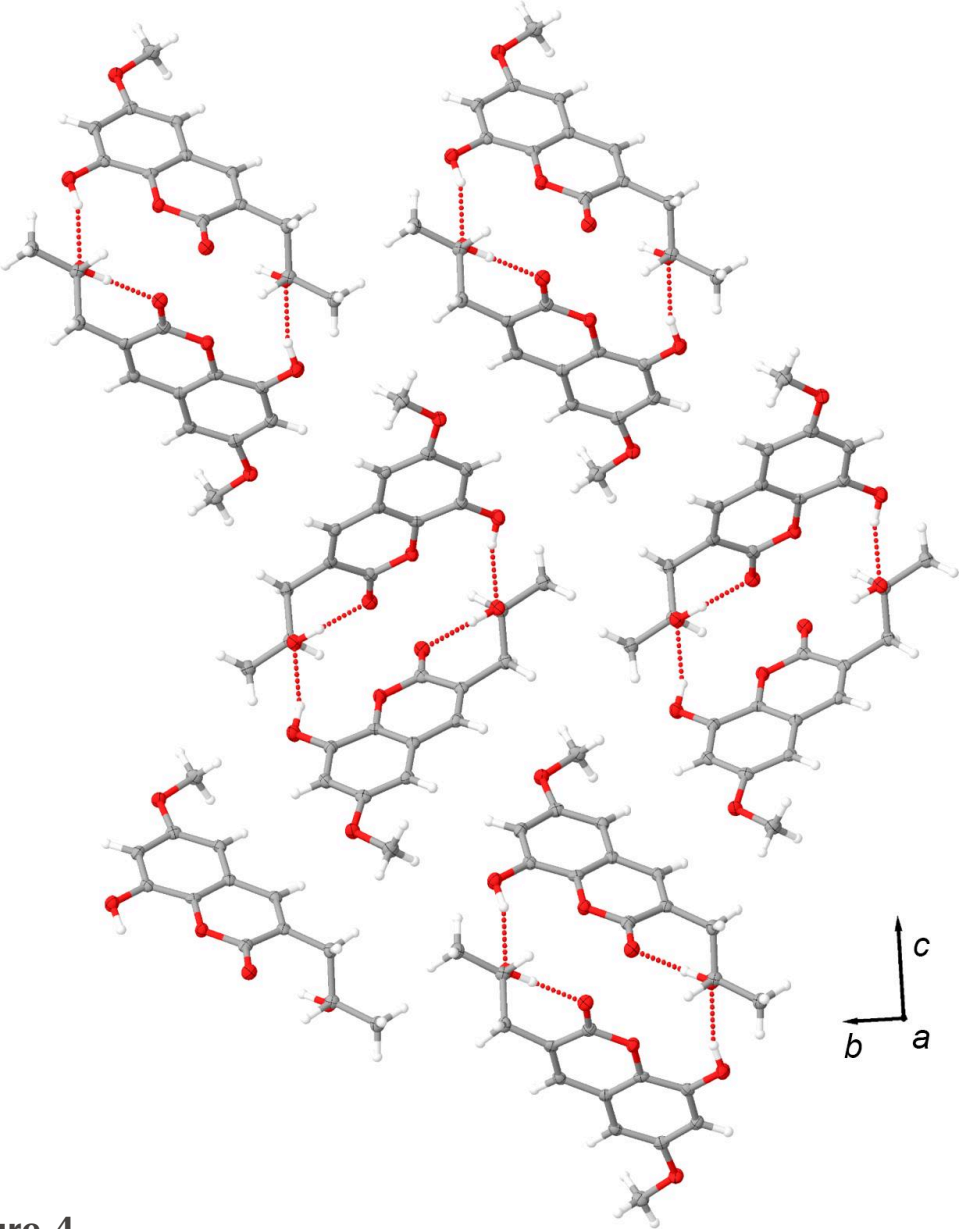
Packing diagram of Berkecoumarin as viewed down the crystallographic *a* axis.

Together we suspect this is what ultimately leads to the needle morphology of the crystals, as evaluations of packing diagrams highlight minimal strong inter­molecular inter­actions between adjacent helical columns (Fig. 4 ▸). The inter­action that is most striking is a C—H⋯O hydrogen bond from the methyl ether group to the phenol O atom; the C13—H13*B*⋯O3(−*x* + 1, *y* − 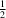 , −*z* + 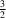 ) hydrogen-bond parameters are 2.60 Å and 162.1°. The distance between the H and O atoms is less than the sum of the van de Waals radii, with an angle greater than 130°. This inter­action is categorized as strong according to the parameters put forth by Johnson and co-workers (Fargher *et al.*, 2022 ▸). Besides this inter­action, there are minimal additional inter-column inter­actions.

### Absolute structure determination analysis from X-ray data

From the X-ray diffraction data, we have determined the Flack parameter to be 0.01 (11) (Parsons *et al.*, 2013 ▸) (Table 1 ▸). Calculation of the Friedif(Cu) value (36) suggests that the *u* value ob­tain­ed here is about the best we could obtain given the chemical make-up of Berkecoumarin and the use of Cu *K*α radiation (Flack & Shmueli, 2007 ▸; Flack, 2008 ▸). The standard uncertainty (*u*) (0.11) is on the edge of what is considered to be acceptable for an established enanti­opure compound (Flack & Bernardinelli, 2000 ▸, 2008 ▸). While the *u* value obtained is 0.01 units beyond the recommendation, we feel confident that we have determined the proper enanti­omer. One reason is that chiral natural products are often produced in an optically pure form and cases of generating enanti­omeric or scalemic products are rare (Finefield *et al.*, 2012 ▸). Furthermore, analysis of the absolute structure using like­li­hood methods (Hooft *et al.*, 2008 ▸) also supports the assignment, with a Hooft parameter of 0.02 (0.9). Finally, the probability statistics indicate that the absolute configuration has been correctly assigned, with a *P*2(true) value of 1.00.

### Absolute structure determination from electron-diffraction data

There is no anomalous dispersion for electron-diffraction data, so determination of the enanti­omer is not possible with a kinematical refinement of the data. However, dynamical refinement has proven to be a powerful and reliable method for determining the absolute configuration of chiral mol­ecules (Brázda *et al.*, 2019 ▸; Klar *et al.*, 2023 ▸; Palatinus, Petříček *et al.*, 2015 ▸; Palatinus, Corrêa *et al.*, 2015 ▸).

Three data sets were imported in *JANA2020*. The model obtained from the X-ray refinement was used as a starting model, although the structure could also be solved by *ab initio* methods directly from the MicroED data. A wedge-shaped crystal model was used to model the thickness variation (Palatinus, Petříček *et al.*, 2015 ▸). The refinement proceeded smoothly, and the refinement statistics are summarized in Table 2 ▸. The overall *R*1(obs) value calculated on all three data sets is 12.82%. This is a relatively large number for dynamical refinement (likely attributable to the high mosaicity of the samples), but it can still be considered acceptable.

The absolute structure was determined by a method described previously (Klar *et al.*, 2023 ▸). Once the refinement of the *S*-enanti­omorph was finalized, an inverted model was created, and, without changing any parameters, it was also refined with the dynamical refinement approach. The correct enanti­omorph can usually be determined directly by com­paring the *R* values of the two refinements. In the current case, the *R* values of the *S*-enanti­omer model are clearly lower than those of the *R*-enanti­omer (Table 4 ▸). The reliability of this qualitative assessment can be qu­anti­fied by the z-score method (Klar *et al.*, 2023 ▸), which provides the confidence level of the hypothesis that one of the enanti­omorphs is the correct one. The results in Table 4 ▸ show that each of the three data sets alone provides statistically significant evidence for the *S*-enanti­omorph (z-score larger than 3). The combined z-score calculated from all three data sets is 6.39, which corresponds to the probability of an incorrect absolute structure assignment of <10^−6^. The absolute structure is thus unambiguously determined.

## Conclusion

Here we have reported the absolute structure configuration of Berkecoumarin, a natural product isolated from extremophilic microbes living in a toxic mining pit lake in Butte, Montana. The chemical make-up of this light-atom mol­ecule pushes the limits of a routine in-house X-ray diffraction absolute structure determination from anomalous scattering. A combination of Flack and Hooft parameters, and probability statistics, indicate the *S*-enanti­omer. To further support this finding, MicroED data were collected, and dynamical refinement was conducted. Despite the high mosaicity and low com­plete­ness, the dynamical method was able to determine the absolute configuration as the *S*-enanti­omer as well, further confirming the assignment. Overall, this work further demonstrates that dynamical refinement of MicroED structures is a powerful and robust method for the absolute structure elucidation of light-atom chiral mol­ecules.

## Supplementary Material

Crystal structure: contains datablock(s) I, global. DOI: 10.1107/S2053229624003061/yp3233sup1.cif

Structure factors: contains datablock(s) I. DOI: 10.1107/S2053229624003061/yp3233Isup2.hkl

CIF file for the Dynamical Refinement of the MicroED data. DOI: 10.1107/S2053229624003061/yp3233sup3.txt

Supporting information file. DOI: 10.1107/S2053229624003061/yp3233Isup4.cml

CCDC references: 2337933, 2331641

## Figures and Tables

**Table 1:** Experimental details

Crystal data	
Chemical formula	C_13_H_14_O_5_
*M*_r_	250.24
Crystal system, space group	Orthorhombic, *P*2_1_2_1_2_1_
Temperature (K)	100
*a*, *b*, *c* (Å)	4.9524 (2), 11.0302 (4), 20.9007 (7)
*V* (Å^3^)	1141.72 (7)
*Z*	4
Radiation type	Cu *K*α
μ (mm^−1^)	0.95
Crystal size (mm)	0.54 × 0.04 × 0.02

Data collection	
Diffractometer	Bruker D8 VENTURE DUO
Absorption correction	Multi-scan (*SADABS*; Krause *et al.*, 2015 ▸)
*T*_min_, *T*_max_	0.547, 0.751
No. of measured, independent and observed [*I* > 2σ(*I*)] reflections	9121, 1575, 1463
*R*_int_	0.053
θ_max_ (°)	57.8
(sin θ/λ)_max_ (Å^−1^)	0.549

Refinement	
*R*[*F* ^2^ > 2σ(*F* ^2^)], *wR*(*F* ^2^), *S*	0.027, 0.067, 1.07
No. of reflections	1575
No. of parameters	173
H-atom treatment	H atoms treated by a mixture of independent and constrained refinement
Δρ_max_, Δρ_min_ (e Å^−3^)	0.14, −0.20
Absolute structure	Flack *x* determined using 555 quotients [(*I* ^+^) − (*I* ^−^)]/[(*I* ^+^) + (*I* ^−^)] (Parsons *et al.*, 2013 ▸)
Absolute structure parameter	0.01 (11)

Computer programs: *APEX4* (Bruker, 2021 ▸), *SAINT* (Bruker, 2015 ▸), *SHELXT2018* (Sheldrick, 2015*b* ▸), *SHELXL2019* (Sheldrick, 2015*a* ▸), and *OLEX2* (Dolomanov *et al.*, 2009 ▸).

**Table 2:** MicroED processing and dynamical refinement experimental details

Experimentation information		
Collection method	Continuous-rotation data collection from three crystals	
Tilt ranges and step^*a*^	Data set	α_min_, α_max_, Δα (°)
	1	−33.34, 34.15, 0.444
	2	−20.46, 17.33, 0.444
	3	−16.02, 27.93, 0.444
Exposure time (ms)	222	
Beam diameter (nm)	600	
Camera length (mm)	788.2	
		
Crystal information		
Empirical formula	C_13_H_14_O_5_	
*Z*, *Z*′	4, 1	
Space group	*P*2_1_2_1_2_1_	
*a*, *b*, *c* (Å)	4.99 (5), 11.22 (5), 21.23 (17)	
Apparent mosaicities (°)	0.48, 0.17, 0.35	
Completeness (%)	65.2	
sin (θ_max_)/λ (Å^−1^)	0.55	
*N*_obs_, *N*_all_	2551, 4111	
Refined parameters	145	
*R*(obs), *mR*(obs)^*b*^ (*I* > 3σ; %)	12.82, 9.49	
*R*(all), *mR*(all)^*b*^ (%)	17.73, 12.23	
*wR*(all), *mwR*(all)^*b*^ (%)	12.80, 9.33	

Notes: (*a*) range of usable frames, not the entire recorded range. (*b*) The dynamical refinement proceeds against unmerged data and, therefore, the *R* and *wR* values are calculated on unmerged data. Therefore, the *mR* and *mwR* are also reported. These values are calculated on the merged data (Klar *et al.*, 2023 ▸).

**Table 3:** Hydrogen-bond geometry (Å, °)

*D*—H⋯*A*	*D*—H	H⋯*A*	*D*⋯*A*	*D*—H⋯*A*
O3—H3⋯O5^i^	0.88 (4)	1.87 (4)	2.723 (3)	161 (4)
O5—H5⋯O1^ii^	0.93 (4)	2.00 (4)	2.915 (3)	170 (4)

Symmetry codes: (i) 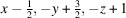 ; (ii) 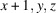 .

**Table 4:** Absolute structure determination by the dynamical refinement Values of z-score above 3 indicate, in a statistically significant manner, that the corresponding enanti­omorph is the correct one.

Data set	*wR*(all) (Enanti­omer *S*)	*wR*(all) (Enanti­omer *R*)	z-score for Enanti­omer *S*
1	15.15	16.69	3.78
2	11.82	12.96	3.81
3	12.30	13.80	3.51
Combined	**12.87**	**14.26**	**6.39**
